# Correction Strategy of Mortars with Trajectory Correction Fuze Based on Image Sensor

**DOI:** 10.3390/s19051211

**Published:** 2019-03-09

**Authors:** Rupeng Li, Dongguang Li, Jieru Fan

**Affiliations:** Science and Technology on Electromechanical Dynamic Control Laboratory, Beijing Institute of Technology, Beijing 100081, China; lirupeng@bit.edu.cn (R.L.); fanjieru@bit.edu.cn (J.F.)

**Keywords:** trajectory correction, image senor, flight mechanics, correction strategy

## Abstract

For a higher accuracy of projectiles, a novel trajectory correction fuze is proposed. In this design, the sensor and actuator were reduced to achieve a balance between performance and affordability. Following introduction of the fuze concept, the flight model was presented and the crossrange and downrange components of trajectory response under control were investigated. The relationship between the inertial coordinate system and the detector coordinate system was studied so that the imager feedback could be used to derive the actual miss distance. The deployment time of canards and roll angle of the forward fuze were derived and used as the inputs of the control system in this strategy. Example closed-loop simulations were implemented to verify the effectiveness of the strategy. The results illustrate that the accuracy increase is evident and the proposed correction concept is applicable for terminal correction of mortars.

## 1. Introduction

Trajectory correction fuze is very effective for improving attack accuracy and reducing impact point dispersion of the gun-launched projectiles [1,2,3,4]. Without any modification of the projectile body, the conventional stock ammunition could obtain the capacity of trajectory correction by simply replacing the trajectory correction fuzes. As such, a fuze can improve the operational effectiveness and maximize the use of stockpiles, and it has received much attention. Zhu [5] analyzed the effect of different canards structure on correction ability using ICEM software. Wu [6] studied the angular motion characteristics during the trajectory correction. Wernert [7] investigated the effect of the canard deployment angle on the stability of the projectiles. Li [8] studied the aerodynamic characteristics for a two-dimensional trajectory correction fuze. Hainz [9] investigated the linear flight model of the projectile under trajectory correction. Chang [10] proposed a simplified projectile swerve solution for trajectory correction. 

The correction strategy design is the foundation for trajectory correction fuze. No matter what correction strategy is adapted, the relevant sensors’ feedback would be necessary. Generally, the GPS receiver is a common way to obtain the projectile position, while gyroscopes and accelerometers may be used to measure the projectile attitude. For a projectile with precise fixed-point initiation, a depth sensor [11] is required. If a classic proportional navigation guidance is considered, a goniometer is needed to track the line of sight (LOS) [12,13]. Additionally, a suitable filtering algorithm may be helpful in information processing during the positioning and tracking. Orton and Marrs [14] used particle filters to track with out-of-sequence measurements with arbitrary lag. Based on that, Martino [15] investigated the application of group importance sampling in particle filtering during the signal processing. In previous research, impact point prediction [16,17,18,19] and model trajectory tracking [20,21,22] are the two most frequently used strategies for trajectory correction fuze. When using the two methods, the target coordinates or the ideal trajectory in the inertial coordinate system are loaded pre-flight. During the flight, the predicted impact point or the projectile states are calculated in real time. By comparison of the target coordinates or ideal trajectory, the difference is formed and used to be the input of the control commander.

The control actuator is another indispensable part of trajectory correction. The common actuators for trajectory correction fuzes are nose-mounted canards [23] and jet thrusters [24]. Compared with the jet thrusters, the canard actuator has a lower cost and needs less modifications for original projectiles. Moreover, because of the restriction of propellant, jet thrusters are difficult to integrate into the fuze. Therefore, the canard actuator is more suitable for trajectory correction fuze. To complete both the crossrange and downrange correction, two pairs of canards are always involved in a general canard actuator [10]. In addition, the deflection angle of the canard is often designed as variable so that the induced aerodynamic correction force can be adjusted according to the needed correction distance [25].

The subject investigated in this research is the mortar, which is frequently used for artillery. Because it is usually launched from a smooth bore gun, it is always regarded as a non-spinning projectile. Recent years the artillery have been seeking a higher striking accuracy when using mortars to attack the target, to shorten operational time and reduce the operational loss. However, the accuracy improvement for current trajectory correction fuze has a limit because real-time information of the target is not involved in the mentioned strategies. The objective of this paper is to further improve operational effectiveness. Therefore, an image sensor was considered in the design of the trajectory correction fuze as it can provide the real position information of the target. To achieve this goal, the accompanying technical challenge should be overcome at the beginning. 

Generally speaking, the space available and updated cost are limited for trajectory correction fuzes used for mortars, so there are some constraints on the inner components of the fuze such as sensors and actuators. Once the imager is used in trajectory correction fuze, the front-end space would be occupied, which would bring about more space resource constraints. Therefore, the balance between performance and affordability of the fuze should be achieved. An overall design with reduced sensors and actuators would be considered. Additionally, for the limitation of trajectory curve and limited detection distance, target detection works only in descent trajectory. The time to go for correction is shorter than that of other correction types. Consequently, the complexity of the correction actuator should be minimized to ensure a rational utilization of space resources and a rapid response. Finally, a suitable corresponding terminal correction strategy based on image sensor with limited feedback information is necessary.

In this paper, we propose a novel design trajectory correction fuze used for mortar, in which the imager is used to provide the target information and no additional sensor is required. The control actuators were reduced and the two-dimensional correction were completed by only a pair of canards and a single-axis motor. For further minimization of actuator complexity, a fixed deflection angle was designed. The correction strategy based on this new trajectory correction fuze was investigated. In this strategy, the information of image sensor provides the miss distance between the target and the projectile, and it is the only feedback used. Based on the newly designed fuze, the deployment time of the canards and the roll angle of the forward fuze were the inputs of the control system. Importantly, during the correction, the canard deflection no longer needed to be adjusted in time. The control complexity was reduced correspondingly, which is suitable for terminal correction with limited time to go.

The paper is organized as follows: In Section 2, the details of the novel trajectory correction fuze are introduced. The flight model under control is constructed. In Section 3, we analyze the trajectory response of the controlled projectile. In Section 4, the correction strategy based on the feedback of the image sensor is proposed. Then, the example simulations are implemented to verify the effectiveness of the strategy. The conclusion is presented in Section 5.

## 2. Flight Dynamic Model 

### 2.1. Trajectory Correction Fuze Concept

The newly designed fuze consists of two parts, which are marked as aft part and forward part, respectively. As illustrated in Figure 1, the aft part is shown in green, in which the safety and arming system, power supply, motor, and computing module are involved. The outside threads are used to provide a stable connection between the projectile body and the aft part, and there is no relative movement between them. The forward part, which is connected to the aft part, is shown in purple. It can rotate dependently relative to the aft part by means of a pair of rolling bearings. The blue mechanism is the canard, which is used to generate an aerodynamic control force for trajectory correction. The canard is attached to the surface of the forward part when there is no control command input. It is designed as a waffle style to increase the windward area when it is exposed to the wind. The reason for this design is that the correction based on imager feedback is only applicable in descent trajectory when the ground target is considered. So, the time left for correction is limited. The design can improve aerodynamic control force by the increased area and ensure a successful correction. The white nose of the fuze represents the image sensor, which is used to detect the target and provide its position information relative to the projectile in the detector coordinate system.

The details of the forward fuze are shown in Figure 2. It should be noted that both the canards and imager are in strapdown with the forward fuze. That means, the canards and imager would rotate at the same frequency and direction with the forward fuze all the time, and the rotation is completely independent of the aft fuze and projectile body. The red part is the mentioned rolling bearings which ensure the relative rotation. The yellow part represents the motor shaft and a pair of internal gears, and the outer gear is fixed to the internal surface of the forward fuze. This mechanism is used to transmit the driving moment from the motor in aft fuze. 

The following two prerequisites should be guaranteed in this fuze design:

(1) The imaging plane of the image sensor should be perpendicular to the longitudinal axis of the projectile, and the imaging plane center should be located on the longitudinal axis.
(2) As illustrated in Figure 2, the dashed line represents the connection between the installation positions of the two canards. We should ensure that this line is parallel to the horizontal axis of image detector, which is illustrated as *x* axis in Figure 2. Additionally, the line, the horizontal axis, and the projectile centroid should be located in the same transverse section of the projectile.

Each inner grid of the canard has a fixed deflection angle. So, the aerodynamic control force is generated once the canards are unfolded, as represented in Figure 2 when the projectile flies under control. The magnitude of induced control force is regarded as constant in terminal trajectory due to the design of fixed deflection angle. Because of the pre-installation requirements mentioned above, the force direction is in line with the positive vertical axis (*y* axis in Figure 2).

The fuze works as follows: The algorithm of correction strategy is loaded in the projectile-borne computer prior to launch. When the projectile passes through its flight apogee and enters the descent trajectory, the image sensor begins to seek the target at a predetermined time. The trajectory deviation can be obtained by the sensor’s feedback. The projectile-borne computer calculates the real deviation in the inertial coordinate system and subsequently obtains two inputs of the actuator according to the loaded algorithm: roll angle of the forward fuze relative to the projectile body and the unfolding time for the canards. Then, the motor drives the forward fuze to rotate independently. Because of the strapdown design, the canards rotate the same angle as the imagery sensor and the forward fuze. The canards then unfold and generate an aerodynamic control force, which can directly lead the projectile towards the target and reduce the trajectory deviation.

It can be seen that the actuator is integrated into the fuze, and the complexity of the actuator is greatly reduced. With this design, the two-dimensional correction can be completed simply by the driving of a single axis motor.

### 2.2. Flight Model of the Projectile under Control

To obtain the accurate actuator inputs from different imager feedback, we should investigate the trajectory response under control. In this section, the necessary coordinate system is introduced and the required mathematical flight model is established as a preliminary study.

The inertial coordinate system o-x_N_y_N_z_N_ is first defined: The origin of this system is the launch site, the *x*-axis points to the target along the horizontal line, the *z*-axis points up in vertical plane and the direction of *y*-axis is determined by right-hand rule, points to right in horizontal plane. The definition of the body-fixed coordinate system o-x_A_y_A_z_A_ is as follows: the origin is located at centroid of the projectile, the *x*-axis points to the projectile head along the longitudinal axis, the *z*-axis is perpendicular to *x*-axis and points up, and the *y*-axis is perpendicular to x_A_-o-z_A_ plane and points to the right. As illustrated in Figure 3, these two coordinate systems can be transferred to each other by the three Euler angles (pitch *θ*, yaw *Ψ*, and roll *γ*).

The kinematic and dynamic equations of the projectile are expressed in Equations (1)–(4), in which *x*, *y*, *z* means the component of projectile position in the inertial coordinate system, and *u*, *v*, *w* and *p*, *q*, *r* mean the component of projectile velocity and angular rate respectively in the body-fixed coordinate system. *F_x_*, *F_y_*, *F_z_* in Equation (3) represent the aerodynamic forces component in the body-fixed coordinate system, and *F_yc_*, *F_zc_* represent the control force in the *y*-axis and *z*-axis induced by the canard, and it only takes effect during the terminal correction. *M_x_*, *M_y_*, *M_z_* and *M_yc_*, *M_zc_* in Equation (4) represent the corresponding moments.

(1) $$\left[\begin{array}{c} \dot{x}\\ \dot{y}\\ \dot{z} \end{array}\right]=\left[\begin{array}{ccc} \cos\theta \cos\psi & -\sin\psi & \sin\theta \cos\psi \\ \cos\theta \sin\psi & \cos\psi & \sin\theta \sin\psi \\ -\sin\theta & 0 & \cos\theta \end{array}\right]\left[\begin{array}{c} u\\ v\\ w \end{array}\right]$$

(2) $$\left[\begin{array}{c} \dot{\gamma }\\ \dot{\theta }\\ \dot{\psi } \end{array}\right]=\left[\begin{array}{ccc} 1 & 0 & \tan\theta \\ 0 & 1 & 0\\ 0 & 0 & 1/\cos\theta \end{array}\right]\left[\begin{array}{c} p\\ q\\ r \end{array}\right]$$

Equations (1) and (2) are kinematic equations used to describe the projectile position and attitude relative to the inertial coordinate system. Specifically, Equation (1) depicts the motion of the projectile centroid. Equation (2) depicts the projectile rotation around its centroid.

(3) {u˙v˙w˙}={FxmFymFzm}+g{−sinθ0cosθ}+{0FycmFzcm}+{0r−q−r0−rtanθqrtanθ0}{uvw}

(4) $$\left\{\begin{array}{c} \dot{p}\\ \dot{q}\\ \dot{r} \end{array}\right\}=\left[I^{-1}\right]\left\{\left\{\begin{array}{c} M_{x}\\ M_{y}\\ M_{z} \end{array}\right\}+\left\{\begin{array}{c} 0\\ M_{yc}\\ M_{zc} \end{array}\right\}-\left[\begin{array}{ccc} 0 & -r & q\\ r & 0 & r\tan\theta \\ -q & -r\tan\theta & 0 \end{array}\right]\left[I\right]\left\{\begin{array}{c} p\\ q\\ r \end{array}\right\}\right\}$$

Equations (3) and (4) are dynamic equations. Similarly, Equation (3) is the dynamic equation of centroid motion. Equation (4) is the dynamic equation of rotation around the centroid.

The aerodynamic force is composed of drag and lift, and the aerodynamic moment is composed of static moment and damping moment. The detailed expressions for aerodynamic force and moment are omitted here for brevity but may be found in the literature [26,27]. For spin-stabilized projectile, Magnus moment is also a component of the aerodynamic moment, and it is induced by high speed rotation. In this investigation, with mortar being investigated object, Magnus moment is not taken into consideration because mortar is always launched from smoothbore guns and has little rotation.

In addition, the relationship between the detector coordinate system and the body-fixed coordinate system is a necessary supplement. When the projectile flies without any control force, the horizontal and vertical axis are parallel to axis *y_A_* and *z_A_*, and if the correction begins and the forward fuze starts to rotate, the detector rotates the same rate due to the strapdown design. As shown in Figure 4, there would be a roll angle *γ_C_* between these two coordinate systems, and their relationship is expressed in Equation (5).

(5) $$\left[\begin{array}{c} x_{D}\\ y_{D} \end{array}\right]=\left[\begin{array}{cc} \cos\gamma_{C} & -\sin\gamma_{C}\\ \sin\gamma_{C} & \cos\gamma_{C} \end{array}\right]\left[\begin{array}{c} y_{A}\\ z_{A} \end{array}\right]$$

## 3. Analysis on Trajectory Response

The trajectory correction fuze does not have sufficient sensors to provide the information of projectile attitude or track its real-time response under control because of the constraint on cost and space. Therefore, for an effective correction, the trajectory shift response under control should be investigated preflight. Once the target, projectile type, and meteorological condition is determined, the aerodynamic parameters, characteristic parameter, muzzle velocity, and elevation are acquired subsequently. With expressions Equations (1)–(4) in Section 2, the ideal trajectory can be computed. A certain mortar is taken as an example to illustrate the response. The physical properties of the example projectile are shown in Table 1, and the meteorological conditions and initial launch conditions are shown in Table 2.

The result of trajectory computation is expressed in Figure 5. The black line means the ballistic trajectory in ideal conditions, and it is defined as ideal trajectory in this research. The downrange distance reaches almost 5043 m, the total flight time is 38.472 s and there is no shift because of the ideal launch condition and the neglect of perturbation. Then, the control force induced by canard is exerted to the projectile in four directions respectively, which are shown in colored lines. The unfolding time of the canard is 30.472 s, and the magnitude of the control force is 14 N.

When the control force is in horizontal plane of the projectile, the downrange correction ability is almost 104 m, whereas when the control force is in vertical plane, the crossrange correction ability is only 62 m. Because our object in this study is a non-spinning mortar, the vertical force has almost no effect on crossrange ability and vice versa. As illustrated in Figure 5, the same control force in different directions brings about the difference in correction ability. The reason is that the gravity acts on the projectile and causes a trajectory curvature. Euler pitch and yaw are used to describe the angular motion in vertical and horizontal plane, and they are directly related to the longitudinal and transverse positions of the projectile, respectively. Control force corrects the trajectory by changing the pitch or yaw. However, the trajectory curvature inhibits the pitch variation when the vertical force is exerted, so there is more correction ability in crossrange than downrange direction with the same control force.

The pitch variation under a horizontal control force and the yaw variation under the vertical control force with the same magnitude were obtained and compared to verify the theory. As shown in Figure 6, a 14 N control force is exerted at 30.472 s. When it points up, a pitch variation is generated, in which the maximum value is about 11 degrees. The variation converges to about 7 degrees under combination of gravity and the vertical control force. The result is shown in Figure 6a. When the control force points to right, the maximum induced yaw is almost 27 degrees and its steady value is increasing along with time. The result is shown in Figure 6b. It is obvious that the angular variation in horizontal plane is more severe than that in vertical plane when control force with the same magnitude is applied separately. So, as shown in Figure 5, there is more subsequent correction ability in the crossrange direction.

Although correction ability with control force with the same magnitude varies in different directions, any deviations between the target and projectile can be expressed by vertical and horizontal components. Therefore, to overcome this problem, we investigated the crossrange and downrange correction ability under different control force magnitude and deployment time. The results should be fitted or stored in the projectile-borne computing system preflight and used for the correction strategy.

Figure 7 illustrates the crossrange and downrange correction ability under different control force (from −14 N to 14 N) and different duration (from 0 s to 8.472 s) for the example projectile, in which the maximum control force induced by canard was set as 14 N, and the maximum duration for correction was set as 8.472 s.

It is remarkable that the flight mechanics and trajectory response under control of different kinds of projectiles varies a lot. However, once the operational conditions and target information are determined, the projectile type and launching conditions are determined subsequently. Therefore, its ideal trajectory can be computed. Based on that, the crossrange and downrange correction ability can be obtained and used to determine the roll angle of the forward fuze and the deployment time of the canard.

## 4. Correction Strategy Design

### 4.1. Determination of Control Parameters

The horizontal and longitudinal component of the total control force in the body-fixed coordinate system is distributed by the roll angle *γ_C_* of the forward fuze. The correction duration is determined by the unfolding time of the canard. Therefore, the trajectory correction is achieved by controlling the roll angle of forward fuze and the unfolding time of canard in this design.

Figure 8 illustrates the deviation between the target and predicted impact point. The deviation is expressed in the inertial coordinate system, and *D_Nx_*, *D_Ny_*, *D_Nz_* represent deviation components of the three coordinate axes. For a successful terminal correction, *D_Nx_*, *D_Ny_* should be reduced to an effective damage scope. Because the position information from the image sensor is based on the body-fixed coordinate system, a transformation equation is proposed in Equation (6), in which *D_Ax_*, *D_Ay_*, *D_Az_* are deviation components in the body-fixed coordinate system. It should be noted that our investigated object is mortar, which can be regarded as a non-spinning projectile. When a projectile with rotation is considered, a magnetic sensor [28,29,30] may be necessary for the transformation.

(6) $$\left[\begin{array}{c} D_{Ax}\\ D_{Ay}\\ D_{Az} \end{array}\right]=\left[\begin{array}{ccc} \cos\theta \cos\psi & \cos\theta \sin\psi & -\sin\theta \\ -\sin\psi & \cos\psi & 0\\ \sin\theta \cos\psi & \sin\theta \sin\psi & \cos\theta \end{array}\right]\left[\begin{array}{c} D_{Nx}\\ D_{Ny}\\ D_{Nz} \end{array}\right]$$

The target position information relative to the projectile in the detector coordinate system is shown in Figure 9. Equation (7) is proposed to build a bridge between the deviation shown in the image sensor and that in the body-fixed coordinate system, in which *f* is the focal length of detector lens, *D_Dx_*, *D_Dy_* are the horizontal and vertical deviation in the detector coordinate system. The relationship between the imager feedback and the required correction ability can be obtained by integrating Equation (6) and Equation (7). That means that the real deviation between the target and the predicted impact in the inertial coordinate system can be obtained by deviation in the detector coordinate system.

(7) $$\left[\begin{array}{c} D_{Dx}\\ D_{Dy} \end{array}\right]=\frac{f}{D_{Ax}-f}\left[\begin{array}{c} D_{Ay}\\ D_{Az} \end{array}\right]$$

It should be noted that the trajectory of mortar has a large curvature, which may have an influence on the detection and correction. The reason is described with Figure 10, in which *X*_1t_, *Z*_1t_ and *X*_2t_, *Z*_2t_ represent the downrange and altitude positions at time *t*_1_ and *t*_2_, respectively, in the vertical plane of the inertial coordinate system; *X*_1P_, *X*_2P_ represent the predicted impact point in downrange direction if the projectile flies in a straight line (i.e., along the optic axis of the detector); and *X*_T_ is the target position in this vertical plane. As illustrated in the figure, the projectile can hit the target accurately along its original trajectory without any correction. We took the projectile at time *t*_1_ as an example; however, there is a deviation |*X*_T_ − *X*_1P_| at the moment, and it can be detected by the imager. That means the deviation caused by trajectory curvature would influence the correction strategy. For more accuracy improvement, the deviation caused by trajectory curvature is considered and compensated into *D_Nx_* in Equation (6). The compensation can be estimated preflight using Equations (8) and (9).

(8) $$\left|X_{1}^{P}-X^{T}\right|=\left|X_{1}^{P}-X_{1}^{t}\right|-\left|X_{1}^{t}-X^{T}\right|$$

(9) $$\left|X_{1}^{P}-X_{1}^{t}\right|=\frac{Z_{1}^{t}}{\tan\theta }$$

By comparison of the deviation at time *t*_1_ and *t*_2_, we found that the deviation was reduced along with time. Therefore, compensation may be fitted as a function of flight time and stored in the projectile-borne computing system. The fitted function for the example projectile in Section 3 is shown in Figure 11.

Since the relationship between the deviation in the inertial coordinate system and that in the detector coordinate system is established, and the trajectory response under various conditions is investigated, the two crucial parameters in this strategy—the expected roll angle of the forward fuze and the deployment time of the canard—can be obtained. As mentioned above, when the projectile enters its descent trajectory, the imager begins to detect the target. According to the information in the detector coordinate system, the needed correction ability in downrange direction *D_Nx_*, and crossrange direction *D_Ny_* are computed. Because the trajectory response is investigated and stored preflight, all the vertical control force *F_zc_* and correction duration *t_zc_* that can meet the requirement of downrange correction ability *D_Nx_* can be derived and expressed as

(10) $$f_{1}\left(F_{zc},t_{zc}\right)=D_{Nx}$$

Similarly, the function for crossrange correction ability can be obtained and expressed as

(11) $$f_{1}\left(F_{yc},t_{yc}\right)=D_{Ny}$$

Because the canard structure and its deflection angle are predetermined, the total control force in terminal correction is regarded as a constant *F_total_*. The relationship between the total control force and its components are written with the expected roll angle *θ_C_*, and the expression is shown in Equation (12). According to the trigonometric function, Equation (13) is obtained.

(12) $$F_{zc}=F_{total}\sin\theta_{C}\;F_{yc}=F_{total}\cos\theta_{C}$$

(13) $${\left(\frac{F_{zc}}{F_{total}}\right)}^{2}+{\left(\frac{F_{yc}}{F_{total}}\right)}^{2}=1$$

Additionally, when the canards unfold, the correction duration for crossrange and downrange directions are the same. For a closed solution, a supplementary expression *t_zc_* = *t_yc_* is needed. Combining Equations (10) to (13) and the supplementary expression, the expected roll angle *θ_C_* and correction duration *t_zc_* (or *t_yc_*) can be derived. Because the total flight time *t_i_* is stored preflight, the deploying time *T_D_* can be computed by Equation (14).

(14) $$T_{D}=t_{i}-t_{yc}$$

### 4.2. Example Simulations and Verification

In this section, the example projectile in Section 3 is recalled to verify the effectiveness of the strategy. The relevant physical properties are not repeated here for brevity. We assume the target position in the inertial coordinate system is (5043 m, 0 m, 0 m). The launch parameters such as elevation and muzzle velocity were determined according to the target position and meteorological condition, and the ideal trajectory were derived subsequently. However, the actual trajectory would be influenced by the random disturbance and the projectile may not fall in effective damage scope. In this example simulation, a −0.5 degree error was added to the ideal elevation, a −0.5 degree error was added to the initial yaw, and a 0.5 m/s error was added to the initial velocity. When the target was detected by the image sensor, the needed crossrange correction ability *D_Ny_* and downrange *D_Nx_* were derived by the Equations (6)–(7) in Section 4 and the compensation of trajectory curvature. Ignoring the sensor’s error, *D_Ny_* and *D_Nx_* were obtained and equaled −8 m and 43 m in this simulation. Because the trajectory under control was investigated preflight, all the combinations of required control force and duration that meet the correction requirements Equations (10) and (11) were derived, and they are illustrated as the form of contours in Figure 12.

Combining with the expressions Equations (12)–(14), the expected roll angle and deployment time were derived and equaled 84.18 degrees and 33.40 s respectively. The simulation result is shown in Figure 13, the ideal trajectory which can hit the target accurately is expressed by red line. The actual trajectory with disturbance is expressed as green. As shown, the projectile missed the target during impact. The blue dashed line represents the trajectory with terminal correction strategy. In this example simulation, the crossrange deviation was reduced by 89.6% and the downrange deviation was reduced by 81.7%.

For a more evident verification for the strategy, 100 ballistic replications with initial conditions uncertainty were conducted, and the corresponding results with terminal correction were compared to verify the accuracy improvements. The projectile properties and ideal launch conditions were the same as in Table 1 and Table 2. The standard deviation of the muzzle velocity was 0.5 m/s, and the standard deviation of initial pitch and yaw was 0.5 degrees. The results are shown in Figure 14, where the horizontal axis of the figure represents the crossrange deviation between impact points and target, while the vertical axis represents the downrange deviation. The scattered blue dots represent the ballistic impact points. It can be seen that the absolute value of maximum crossrange and downrange deviation are about 78.9 m and 61.4 m, respectively. The scattered red dots represent the corrected impact points. When the correction strategy was applied, the maximum crossrange deviation was reduced to 11.2 m, while the maximum downrange deviation was reduced to 11.3 m. For further analysis, the definition of circular error probable (CEP) is introduced here: It is defined as the radius of a circle, centered on the target, whose boundary is expected to include the landing points of 50% of the rounds. It is used to measure the precision of a certain ammunition. As illustrated in the figure, the CEP of ballistic impact points is about 40 m, while that of corrected impact points is reduced to about 5 m. An 87.5% accuracy increase is obtained. Therefore, the effectiveness of the novel trajectory correction fuze and strategy is verified.

## 5. Conclusions

A novel trajectory correction fuze based on image senor was proposed, in which the complexity of the actuator was reduced. The two-dimensional correction was completed by a single motor. The unique strapdown structure and pre-designed installation location of the canard ensured an easily implemented correction process. The workflow of this fuze was depicted and relevant correction strategy was investigated. In this strategy, the flight model with terminal control for the selected projectile is established, and the flight mechanics and trajectory response are studied preflight. The transformation relation between the inertial coordinate system and the detector coordinate system was investigated. The needed correction ability was obtained by the imager feedback and could be divided into crossrange direction and downrange direction. The components and duration of the control force were derived according to needed correction ability, and the deployment time of the canards and the roll angle of the forward fuze were subsequently obtained. An example of a disturbed trajectory was used to verify the performance of the correction strategy. The result shows that the crossrange deviation was reduced by 89.6% and the downrange deviation was reduced by 81.7% with the terminal correction. One hundred replication simulations were implemented for further demonstration. The results show that an 87.5% accuracy increase was obtained for CEP.

It should be noted that the fuze and strategy are applicable for mortars and other non-spinning projectiles. When it is extended to scope of spin-stabilized projectiles, an attitude sensor may be used to provide the roll information, and the coupled effect on the trajectory response induced by rotation should be taken into consideration. Additionally, the paper considers a relatively ideal case; further study should be completed in the case of real uncertain aerodynamic coefficients and sensors’ inherent error.

## Figures and Tables

**Figure 1:**
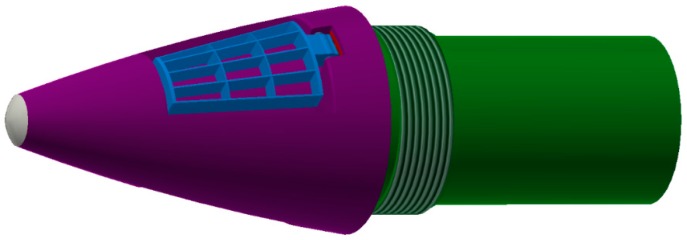
Appearance of the trajectory correction fuze.

**Figure 2:**
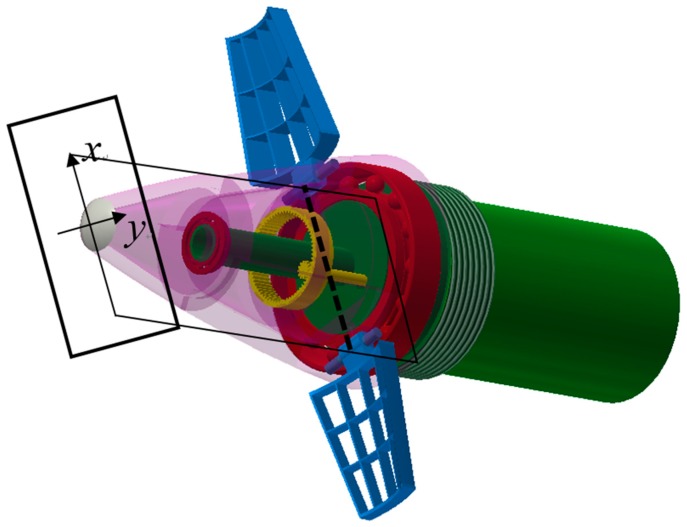
Maneuvering components in forward fuze.

**Figure 3:**
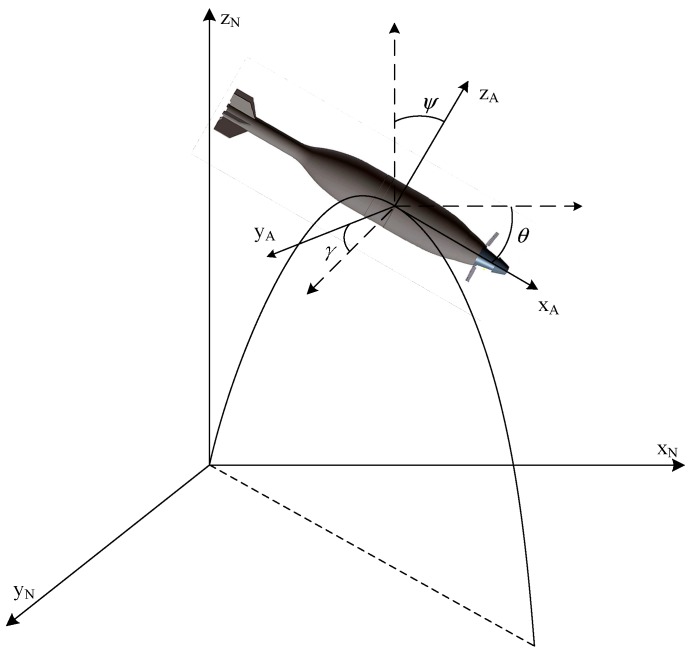
Inertial and body-fixed coordinate systems.

**Figure 4:**
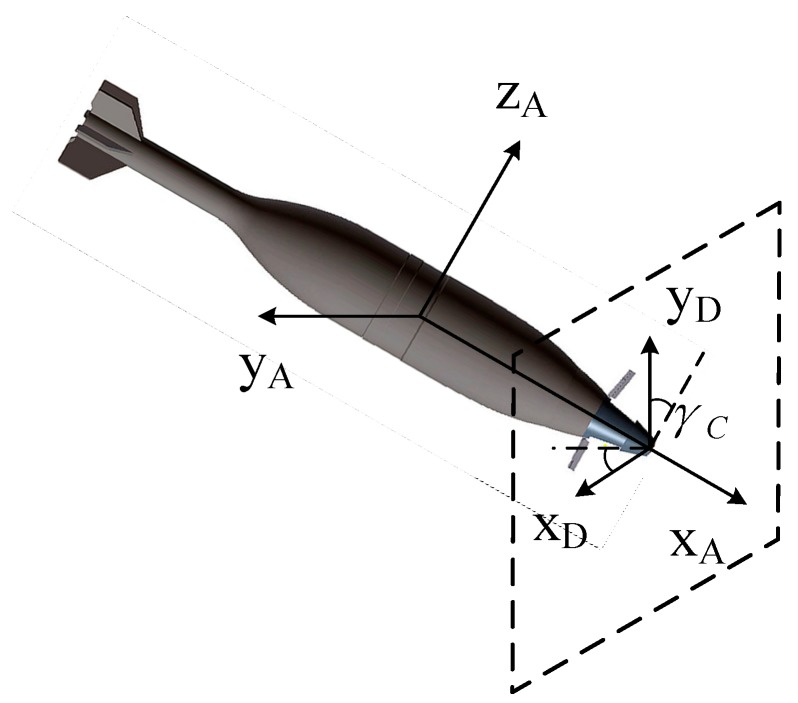
The detector coordinate system.

**Figure 5:**
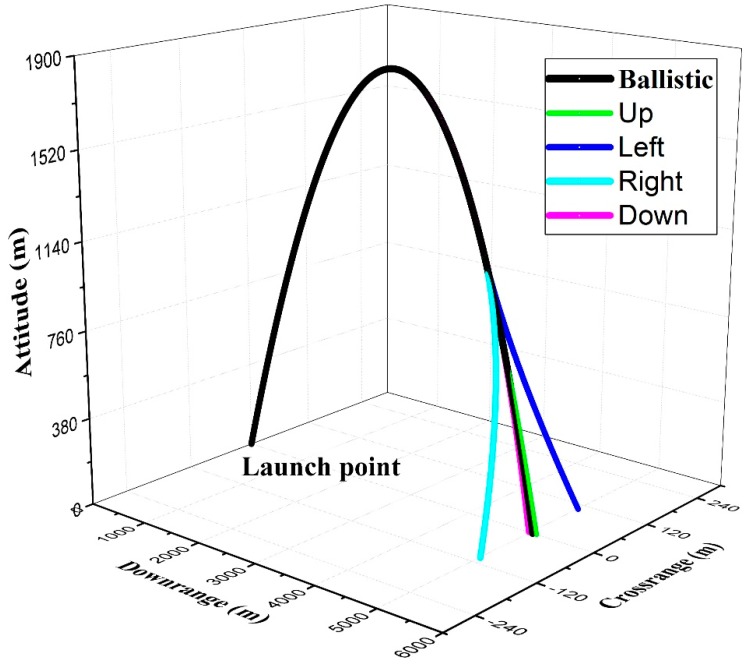
Trajectory of the example projectile.

**Figure 6:**
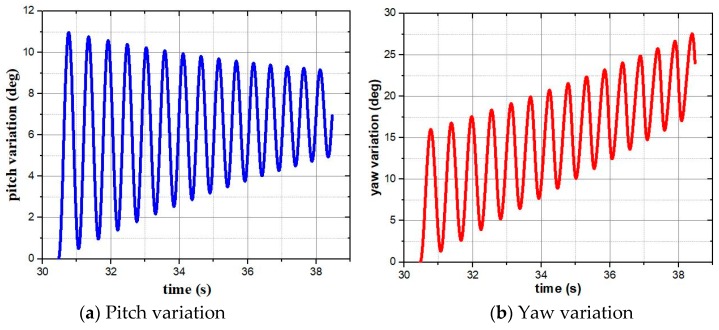
Pitch and yaw variation.

**Figure 7:**
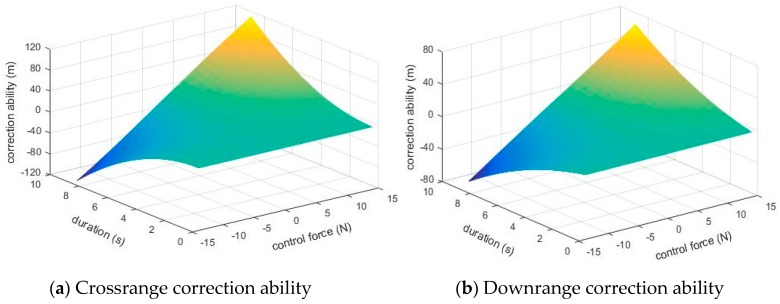
The correction ability with control force and duration.

**Figure 8:**
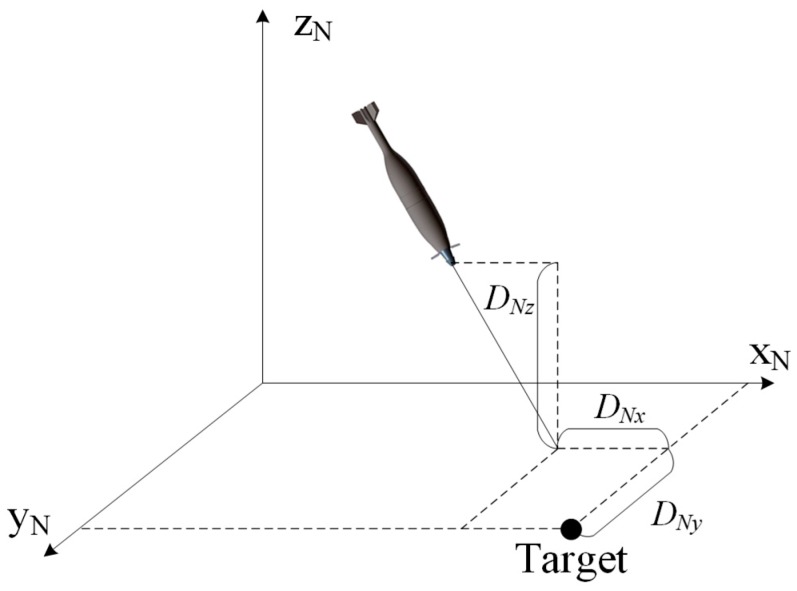
Schematic of the deviation in the inertial coordinate system.

**Figure 9:**
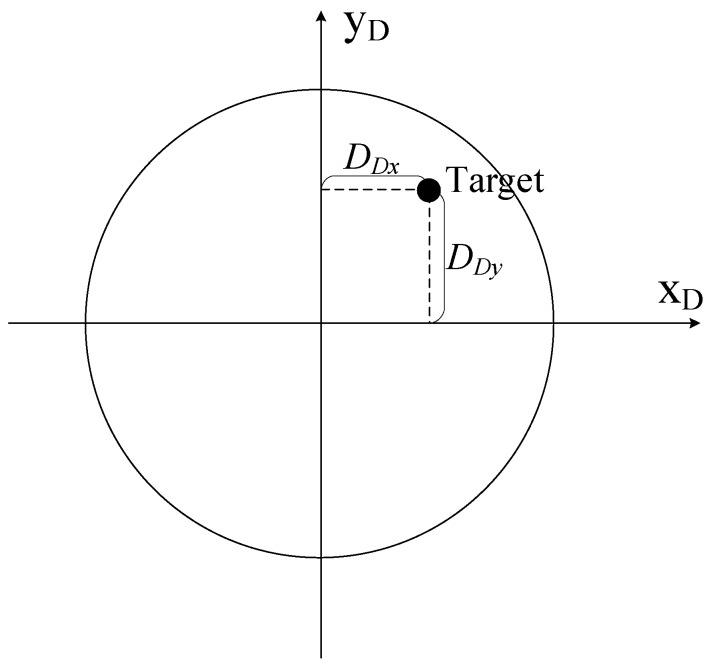
Schematic of the deviation in the detector coordinate system.

**Figure 10:**
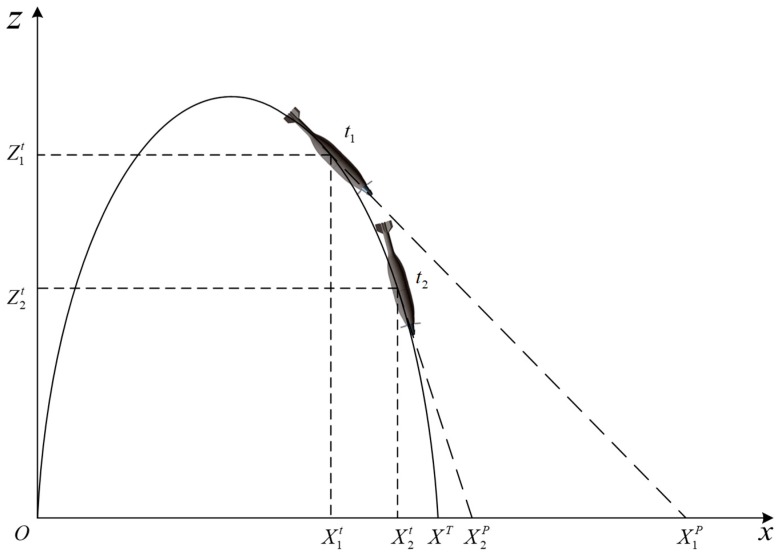
Schematic of the trajectory curvature.

**Figure 11:**
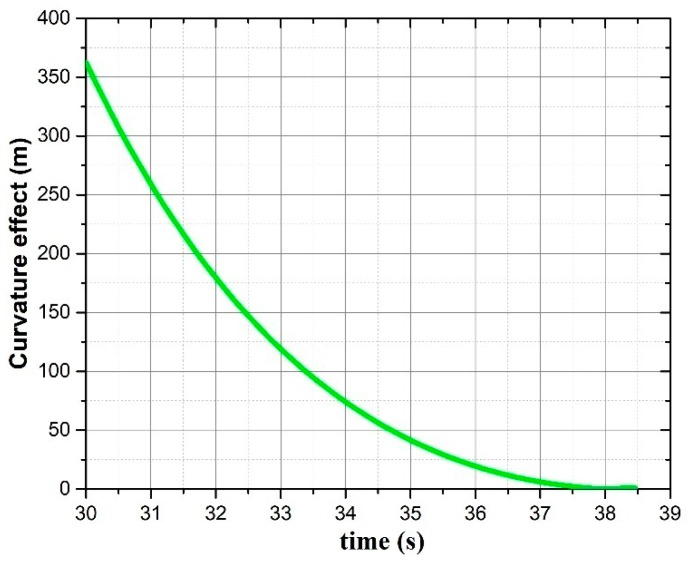
Deviation induced by curvature along with time.

**Figure 12:**
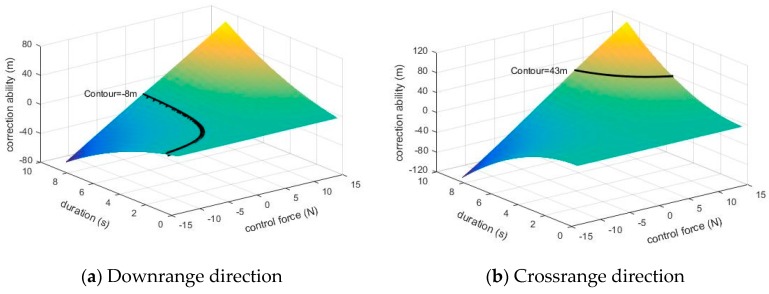
Control forces and durations that meet the correction requirement.

**Figure 13:**
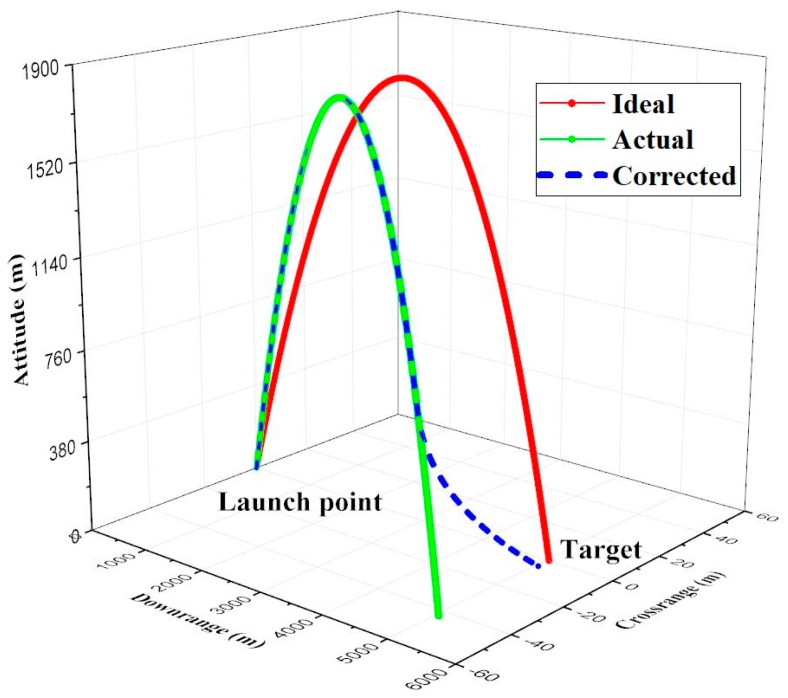
The effectiveness of the correction strategy.

**Figure 14:**
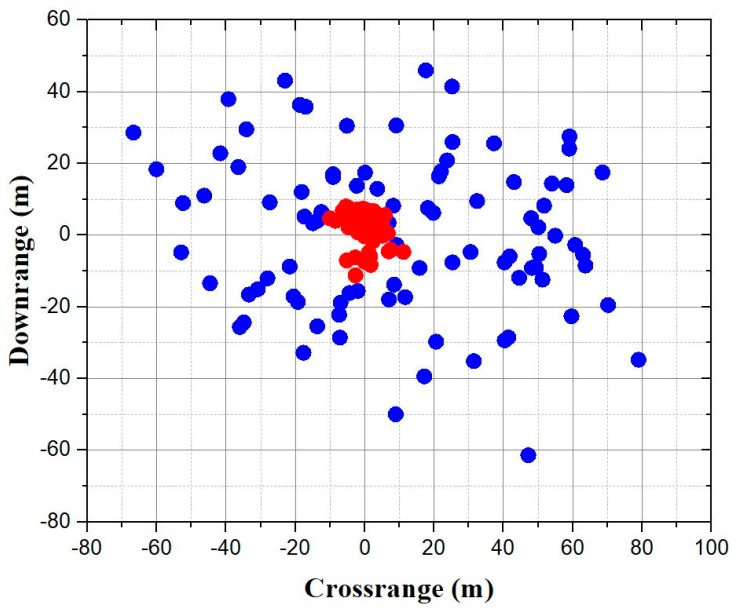
Deviation between impact points and target for ballistic and corrected trajectories.

**Table 1:** Physical properties of the example projectile.

Physical Properties	
Mass (kg)	15.5
Diameter (m)	0.12
Length (m)	0.80
Center of gravity to bottom (m)	0.478
I_xx_ (kg-m^2^)	0.0296
I_yy_ (kg-m^2^)	0.375
I_zz_ (kg-m^2^)	0.375

**Table 2:** Meteorological conditions and initial launch conditions.

Initial Conditions		Meteorological Conditions	
Velocity (m/s)	272	Ground pressure	1000 hPa
Pitch (degree)	53	Virtual temperature	288.9
Direction (degree)	0	Longitudinal wind	0 m/s
Spin-rate (rad/s)	0	Lateral wind	0 m/s
